# Using random forests for assistance in the curation of G-protein coupled receptor databases

**DOI:** 10.1186/s12938-017-0357-4

**Published:** 2017-08-18

**Authors:** Aleksei Shkurin, Alfredo Vellido

**Affiliations:** 1 Department of Computer Science, Universitat Politècnica de Catalunya, C. Jordi Girona, 1-3, 08034 Barcelona, Spain; 2 Technology, Communication and Transport Department, Mikkeli University of Applied Sciences, Patteristonkatu 3, 50100 Mikkeli, Finland

**Keywords:** G-Protein coupled receptors, Machine learning, Random forests, Database curation

## Abstract

**Background:**

Biology is experiencing a gradual but fast transformation from a laboratory-centred science towards a data-centred one. As such, it requires robust data engineering and the use of quantitative data analysis methods as part of database curation. This paper focuses on G protein-coupled receptors, a large and heterogeneous super-family of cell membrane proteins of interest to biology in general. One of its families, Class C, is of particular interest to pharmacology and drug design. This family is quite heterogeneous on its own, and the discrimination of its several sub-families is a challenging problem. In the absence of known crystal structure, such discrimination must rely on their primary amino acid sequences.

**Methods:**

We are interested not as much in achieving maximum sub-family discrimination accuracy using quantitative methods, but in exploring sequence misclassification behavior. Specifically, we are interested in isolating those sequences showing consistent misclassification, that is, sequences that are very often misclassified and almost always to the same wrong sub-family. Random forests are used for this analysis due to their ensemble nature, which makes them naturally suited to gauge the consistency of misclassification. This consistency is here defined through the voting scheme of their base tree classifiers.

**Results:**

Detailed consistency results for the random forest ensemble classification were obtained for all receptors and for all data transformations of their unaligned primary sequences. Shortlists of the most consistently misclassified receptors for each subfamily and transformation, as well as an overall shortlist including those cases that were consistently misclassified *across* transformations, were obtained. The latter should be referred to experts for further investigation as a data curation task.

**Conclusion:**

The automatic discrimination of the Class C sub-families of G protein-coupled receptors from their unaligned primary sequences shows clear limits. This study has investigated in some detail the consistency of their misclassification using random forest ensemble classifiers. Different sub-families have been shown to display very different discrimination consistency behaviors. The individual identification of consistently misclassified sequences should provide a tool for quality control to GPCR database curators.

**Electronic supplementary material:**

The online version of this article (doi:10.1186/s12938-017-0357-4) contains supplementary material, which is available to authorized users.

## Background

Biology in general is experiencing a gradual but nevertheless fast transformation from a laboratory-centred science towards a data-centred one [1]. Such transformation can be seen as the result of the coalescence of at least two drivers: the quick progression of information technology systems capabilities and the not much slower advances on data acquisition methods in the different sub-fields of biology.

All of this is particularly relevant to the *omics* sciences and to bioinformatics. Consistent and concerted efforts are being made in these fields to guarantee the scientific community reliable access to large, heterogeneous and ever-increasing databases. A central concept to the tasks of maintaining and managing such complex databases is that of data curation, which, in the context of biology, is sometimes referred to as biocuration [2].

The objective of the analyses reported in this paper is the large super-family of G protein-coupled receptors (GPCRs). These are eukaryotic cell membrane proteins that are considered of interest to biology in general. Within GPCRs, our specific interest is in one of its families, namely Class C. This family is of particular relevance to current pharmacology, as its members have shown to be therapeutic drug targets that could be involved in the treatment of specific neuro-degenerative diseases such as Alzheimer’s disease, schizophrenia and Parkinson’s disease, amongst others [3], given that most of them are expressed in the central nervous system.

Little is known of the complete crystal (expressing the 3-D) structure of GPCRs and it is only recently that some partial GPCR structures have been solved. They are mostly from Class A [4]. For Class C, instead, no full crystal structure has yet been solved; in fact, only two transmembrane domains and several extracellular domains have been described over the last few years in [5, 6]. This lack of knowledge about their tertiary and quaternary structures means that, faced with the challenge of investigating the functionality of these receptors, we are bound to rely on the analysis of their primary structure, that is, of their amino acid symbolic sequences. This information, fortunately, is publicly available from existing curated databases.

The Class C family is by no means homogeneous and its members have been tagged as belonging to a rich taxonomy of sub-families. Note that many of these are even further sub-divided into types at several levels. This means that the labeling of all these sub-families becomes quite a challenge for the expert and, consequently, any process attempting the automatic sub-family classification unavoidably becomes itself a challenging problem [7]. Again, and as stated earlier in this introduction, such sub-family discrimination becomes a data analysis problem, for which statistics and machine learning approaches can provide well-founded and robust solutions [8].

It must be stressed that, in this study, we are interested less in the problem of achieving maximum sub-family discrimination accuracy (as, for instance, in [7]) than in the exploration of sequence misclassification behavior. We assume that the majority of sequences are adequately characterized (that is, that they are correctly labeled in the database according to sub-family), but we are keen on investigating which sequences are commonly misclassified by computer-based methods and what type of misclassification they suffer. The starting point for these concerns are previously reported results indicating that the discriminatory classification of Class C GPCRs from transformations of their primary sequences shows clear limits [9, 10].

More specifically, we aim to isolate those sequences that show consistent misclassification, in the sense that they are very often misclassified and almost always assigned to the same sub-family (this sub-family being other than the one described by their database label). This is not to be mistaken with sequences that might be misclassified for their partial similarity with different sub-families (what we could call class borderline cases) and which could show far less consistent behavior.

According to this goal, the current study, which is an extension of [11], and its reported experiments aim at assisting the task of database curation by providing data-based evidence of potential GPCR quality control issues. This should be accomplished by identifying and shortlisting cases whose original sub-family assignment is highly questionable from the data modelling results, thus motivating further expert intervention.

Random forests (RF) are the machine learning method of choice in this work for the task of assessing the consistency of (mis)classification. This choice is justified by their ensemble nature, in which the many base classifiers they consist of coalesce in the decision of assigning each given sequence to a class (GPCR sub-family). This *collective decision* process makes them naturally suited to assess the consistency of the classification decisions.

Given that our analyses are based on GPCR primary sequences, a data pre-processing problem obviously arises, which is the choice of transformation of the varying-length sequential symbolic data into formats that are suitable for multivariate data analysis. Such transformations might use the complete unaligned sequences, or methods of multiple sequence alignment. Here, we use a number of unaligned sequence transformations.

## Methods

This section first introduces some basics about the task of curation of protein databases. This is followed by a description of the data used in the reported experiments and an introduction to the RF algorithm as applied to the analysis of these data.

### Curation of protein databases

As mentioned in the introduction, this paper presents a data-based analytical method for the assessment of the consistency of discriminatory classification of sub-families of GPCRs. It is suggested here that such method could be used to assist protein database curators in tasks of data quality control.

Biocuration has become a need in biology due to the exponential growth in data availability in all its many sub-fields and particularly in bioinformatics. It has been described as “the activity of organizing, representing and making biological information accessible” [2] to biologists. Therefore, it should at least partially concern data engineering tasks. Nevertheless, it is far from being an established and well-defined activity and failure to establish and standardize biocuration procedures and to fund these efforts properly would risk the possibility of channeling the data deluge for scientific knowledge extraction.

One of the challenges of curation is the unambiguous identification of biological entities (proteins in the case of the current study) from existing studies and literature. In the end, data trustworthiness can only be ensured through costly data management [12]. This task is uncertain and error-prone, so that the development of computational procedures to assist human experts in it is worth pursuing. Note that GPCRs have been categorized into the Classes A–F (as described in the next sub-section) based on sequence homology procedures [13]. That is, receptor labeling is itself homology model-based and, therefore, uncertain to a degree and at least debatable.

The background for our study is the preliminary evidence suggesting that there seems to exist an upper bound to Class C GPCR discriminability according to the existing sub-family labels [9, 10]. We aim to establish if certain receptors show clear patterns of consistent misclassification and whether this might be the cause for the existence of such upper bound.

### Class C data from the GPCRdb

This study is based on the analysis of data extracted from GPCRdb, a publicly accessible molecular-class information repository for GPCRs [13]. This endeavour was started in 1993 and it is now in its fifth release, with stewardship by David Gloriam’s group at the University of Copenhagen from 2013 and part of the GLISTEN EU COST Action [14] for the creation of “a pan-European multidisciplinary network of researchers investigating G protein-coupled receptor signalling”.

In this repository, the GPCR super-family is divided into major classes following the IUPHAR [15] system, including: A (rhodopsin like), B (secretin and adhesion), C (glutamate), F (Frizzled) and others, based on the ligand types, functions and sequence similarities.

As previously stated, the current study focuses on Class C GPCRs, a quite heterogeneous family that includes seven main sub-families: metabotropic glutamate (mG) receptors, calcium sensing (CS), GABA$_B$ (GB), vomeronasal (VN), pheromone (Ph), odorant (Od) and taste (Ta).

*mG* receptors are activated by glutamate, a major excitatory neurotransmitter in the brain. These receptors are involved in neurological disorders including Alzheimer’s and Parkinson’s diseases, Fragile X syndrome, depression, schizophrenia, anxiety, and pain. Some preliminary information about the binding sites and behavior of two subtypes (1 and 5) of *mG* receptors has been described in [5, 6] from their crystal structures, as summarily reviewed in [16]. The *CS* receptor is activated by the calcium ion and it is known to play a key role in extra-cellular calcium homeostasis regulation. *GB* is a neurotransmitter that mediates most inhibitory actions in the central nervous system; it is involved in chronic pain, anxiety, depression and addiction pathologies. *VN*, *Ph*, *Od* and *Ta* are all involved in physiological roles related to the senses of smell and taste.

A total of 1510 Class C GPCR sequences (from version 11.3.4, March 2011 of the database), belonging to the previously mentioned seven sub-families, were analyzed using the RF models described in the following sub-section. Their distribution of cases by sub-family is displayed in Table 1.

**Table 1:** Number of available sequences in each of GPCR Class C sub-families [26]

Sub-family	Acronym	$\sharp$ sequences
Metabotropic glutamate	mG	351
Calcium sensing	CS	48
GABA$_B$	GB	208
Vomeronasal	Vn	344
Pheromone	Ph	392
Odorant	Od	102
Taste	Ta	65

Given that the primary sequences cannot be analysed as symbolic arrays using standard statistical, pattern recognition and machine learning methods, they have to be transformed for subsequent investigation. Several transformations were considered in our experiments.

The first one uses directly the 20 amino acids of the receptor sequence *alphabet*. An example of this type of transformations is amino acid composition (AAC) [17], in which each sequence is described by the frequencies of appearance of the amino acids. By using only frequencies, AAC ignores the order embedded into the sequential information itself (i.e., the relative position of the amino acids in the sequence). Despite such simplicity, its use has previously yielded surprisingly solid sub-family discrimination results [17, 18].

Subsets of amino acids may share similar physico-chemical properties, which makes them equivalent at a functional level [19]. This equivalence would make them somehow redundant and the use of amino acid groupings based on physicochemical similarity becomes advisable. Amino acid grouping also helps computations by reducing the dimensionality of the analysed data set. For this study, two alternative groupings were used, in the form of sub-sequence frequencies (see Table 2): the Sezerman (SEZ) alphabet (11 groups) [20] and the Davies Random (DAV) alphabet (9 groups) [19]. We select these groupings for analysis because they are the outcome of a selection process and their performance in the classification of GPCRs into their major classes was positively evaluated in [19, 20].

**Table 2:** Amino acid grouping schemes

Grouping	1	2	3	4	5	6	7	8	9	0	X
SEZ	IVLM	RKH	DE	QN	ST	A	GT	W	C	YF	P
DAV	SG	DVIA	RQN	KP	WHY	C	LE	MF	T		

Amino acids and their groupings were not just used as such in this study, but in the form of *n*-grams, which are subsequences of length *n*. The concept of *n*-grams is well-known in protein analysis [21, 22]. Here, we used the relative frequencies of the *n*-grams. Therefore, the *n*-gram representation consists of the relative frequency of each *n*-gram in a sequence (note that for Sezerman and Davies, the length of the *n*-gram is not taken in number of amino acids, but in number of groupings). Due to the exponential growth of the size of *n*-grams, experiments were limited to *n*-grams of size 1, 2 and 3.

### Random forests

Since their definition in the first years of the century, RFs [23] have become a popular and widely-used machine learning tool for classification and regression tasks. This is particularly true in the areas of computational biology and bioinformatics [24]. In these fields, RFs have de facto become standard methods, especially adequate in settings with poor observations-to-variables ratios (of which the current study is a mild case). They are also capable of coping well with highly correlated variables and scale nicely to multi-class problems such as the one investigated here, avoiding the more complex *one-vs-one* or *one-vs-all* classification schemes that are common in the field [9].

**Fig. 1:**
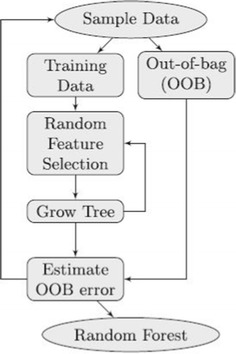
RF scheme. General graphical scheme of the RF algorithm

The general graphical scheme of the RF algorithm is sketched in Fig. 1. At each split of the observed sample data, a random subset of variables is selected (in what is called a random subspace method) and the process is repeated until a specified number of base decision tree classifiers is generated. Each tree is built from a bootstrap sample drawn with replacement from the observed data (the available sample), and the predictions of all trees are finally aggregated through majority voting.

A feature of RFs is the definition of an out-of-bag (OOB) error, which is calculated from observations that were not used to build a particular base decision tree; it can thus be considered as an internal cross-validation error measure [24, 25]. This is an important feature for the type of experiments carried out in this study, because it simplifies the otherwise cumbersome cross-validation procedures that would be required if alternative classification methods such as, for instance, support vector machines or artificial neural networks were used [23].

The fact that RFs are defined as ensemble-of-trees classifiers also means that these models are naturally suited to the task of analysing protein sequence misclassification behavior. The reason for that is that we are interested in methods that naturally work according to a voting scheme in order to assign a sequence observation to a given class (sub-family). These votes from each individual decision tree allow us to more closely inspect the performance of each Class C GPCR sequence. Furthermore, pooling the votes also allows us to gauge the consistency of the (mis)classification results for the sequences of any given sub-family.

## Results and discussion

In this section we first provide details of the experimental settings. This is followed by a presentation of all results and their discussion.

### Experimental settings

As mentioned in the previous section, the subset of sequences belonging to Class C acquired from the GPCRdb database were transformed in three ways: AAC, Sezerman (11 groupings) and Davies (9 groupings). All possible *n*-grams of sizes 1 to 3 were built for each of them and the relative frequencies of the n-grams of size 1, 2 and 3 (for Sezerman and Davies, the length of the n-gram is not taken in number of amino acids, but in number of groupings) were calculated.

In previous research [26], feature selection was performed on these transformed datasets using statistical *t* test filtering to establish a ranking of relevance of the available features. This led to the choice of subsets of features whose test was significant for different numbers of binary classifiers (note that, taking into account that we analyze 7 Class C sub-families, we would have 21 different *one-vs-one* binary classifiers and, at best, a subset of features that was significant for all 21 of them might be obtained).

The subsets of features that achieved the best classification performance in [26] become the starting point for our RF models. Their number of features is 585 for *n*-grams using AAC, 386 for *n*-grams using Sezerman groupings and 238 for *n*-grams using Davies groupings. This makes the discrimination problem a mild case of poor observations-to-variables ratio, as previously indicated.

The RF model was trained using the *randomForest* and *matrixStatsR* packages of the R programming language. To ensure the reproducibility of the results, the random number generator (RNG) state was set to a value of 42. The model was stratified to ensure that the difference in the number of cases between sub-families did not significantly affect the results. It included 500 trees, yielding sufficient performance while keeping computational costs relatively small. On an Apple MacBook Pro with 2.3 GHz Quad-core Intel i7 CPU and 8 Gb RAM, the CPU times for the execution of the processes were 0.028, 0.024 and 0.026 s for selected subsets of *n*-grams generated using AAC, Sezerman and Davies transformations, respectively.

**Fig. 2:**
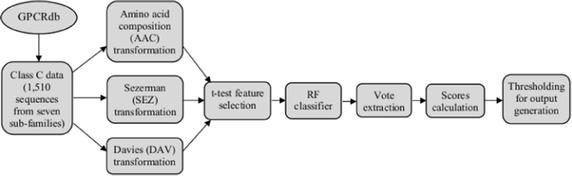
Experimental workflow. Step-by-step graphical representation of the experimental workflow

The whole experimental workflow is graphically summarized in Fig. 2.

### Experimental results

In order to start from a basic reference assessment of performance of the models, overall accuracies (understood as the ratio of correctly classified sequences to all sequences) for all the models were calculated. They are as follows: 0.91 for the selected subset of *n*-grams using AAC; 0.90 for the subset selected using Sezerman transformation and 0.88 for the subset selected using Davies transformation.

Given that the main target is the investigation of the details of the Class C GPCR misclassification behavior, we first calculated the confusion matrices for our RFs. The results are presented in Table 3 for the selected subset of *n*-grams using AAC; in Table 4 for the selected subset of *n*-grams using Sezerman groupings; and in Table 5 for the selected subset of *n*-grams using Davies groupings. All these confusion matrices also include sub-family-specific classification errors (which are equivalent to 1-*sensitivity* for each sub-family). Confusion matrices provide us with an intuitive overall assessment of how scattered the classification results are.

**Table 3:** Confusion matrix corresponding to the RF model for the selected subset of *n*-grams using AAC

	PC							
	mG	CS	GB	VN	Ph	Od	Ta	Class.error
TC								
mG	341	0	4	0	4	2	0	0.028
CS	1	45	1	0	1	0	0	0.062
GB	7	0	201	0	0	0	0	0.033
VN	1	1	0	311	27	3	1	0.096
Ph	14	0	0	24	351	2	1	0.104
Od	4	0	0	6	25	67	0	0.343
Ta	1	0	0	0	0	0	64	0.015

TC *TrueClass*, PC *PredictedClass*

**Table 4:** Confusion matrix corresponding to the RF model for the selected subset of *n*-grams using Sezerman transformation

	PC							
	mG	CS	GB	VN	Ph	Od	Ta	Class.error
TC								
mG	338	0	5	2	4	2	0	0.037
CS	2	43	1	0	2	0	0	0.104
GB	7	0	201	0	0	0	0	0.033
VN	3	0	0	304	33	4	0	0.116
Ph	14	0	2	25	349	2	0	0.109
Od	4	0	0	7	27	64	0	0.372
Ta	1	0	0	0	0	0	64	0.015

TC *TrueClass*, PC *PredictedClass*

**Table 5:** Confusion matrix corresponding to the RF model for the selected subset of *n*-grams using Davies transformation

	PC							
	mG	CS	GB	VN	Ph	Od	Ta	Class.error
TC								
mG	331	0	5	3	9	3	0	0.057
CS	1	43	1	1	2	0	0	0.104
GB	6	0	200	1	1	0	0	0.038
VN	8	0	1	296	34	5	0	0.139
Ph	11	0	2	35	341	3	0	0.130
Od	1	1	1	8	31	60	0	0.411
Ta	2	0	0	0	3	0	60	0.077

TC *TrueClass*, PC *PredictedClass*

The overall accuracy and the confusion matrices are aggregated measures that do not inform about the consistency of individual sequence classifications. We now recall the fact that the sub-family assignments made by the RF model are the result of the individual voting of 500 base trees.

On a first and still sub-family centered approximation, the votes of these 500 trees for each sub-family were extracted and mean values of their voting ratios were calculated as a measure of sub-family classification consistency. For better illustration, only the results for mG, GB and Ph are presented in the main text, in Table 6. These three sub-families are selected as opposite examples of rather well discriminated sub-families (mG and GB) vs. a comparatively poorly discriminated one (Ph). Additionally, the spread of the votes is shown in Table 7 using the standard deviation of the voting ratios.

**Table 6:** Consistencies of the mG, GB and Ph sub-families for the different data sets

	Amino acid						
	*mG*	CS	GB	VN	*Ph*	Od	Ta
*mG*	*0.84*	0.01	0.05	0.02	0.05	0.01	0.02
*GB*	0.09	0.00	*0.87*	0.01	0.02	0.00	0.01
*Ph*	0.05	0.01	0.01	0.18	*0.68*	0.06	0.01

	Sezerman						
	*mG*	CS	GB	VN	*Ph*	Od	Ta
*mG*	*0.81*	0.01	0.05	0.03	0.06	0.002	0.02
*GB*	0.08	0.01	*0.86*	0.01	0.02	0.00	0.01
*Ph*	0.06	0.01	0.01	0.21	*0.63*	0.06	0.02

	Davies						
	*mG*	CS	GB	VN	*Ph*	Od	Ta
*mG*	*0.91*	0.01	0.04	0.04	0.06	0.01	0.02
*GB*	0.08	0.01	*0.84*	0.02	0.03	0.01	0.01
*Ph*	0.06	0.02	0.02	0.19	*0.63*	0.07	0.02

Values in italics are the highest per sub-family

**Table 7:** Vote spread of the mG, GB and Ph sub-families for the different data sets

	Amino acid						
	*mG*	CS	GB	VN	*Ph*	Od	Ta
*mG*	*0.21*	0.02	0.09	0.04	0.07	0.06	0.03
*GB*	0.13	0.01	*0.17*	0.02	0.03	0.01	0.01
*Ph*	0.12	0.02	0.04	0.14	*0.21*	0.08	0.03

	Sezerman						
	*mG*	CS	GB	VN	*Ph*	Od	Ta
*mG*	*0.22*	0.02	0.08	0.05	0.08	0.05	0.03
*GB*	0.12	0.01	*0.18*	0.02	0.03	0.01	0.01
*Ph*	0.11	0.02	0.04	0.13	*0.19*	0.07	0.03

	Davies						
	*mG*	CS	GB	VN	*Ph*	Od	Ta
*mG*	*0.24*	0.02	0.09	0.06	0.09	0.06	0.03
*GB*	0.11	0.02	*0.20*	0.04	0.05	0.01	0.02
*Ph*	0.11	0.03	0.05	0.16	*0.21*	0.08	0.03

Values in italics are the highest per sub-family

On a second level of detail, the consistency of RF voting for each sequence in the analyzed receptors was calculated. A consistency of 100% can only be reached when the 500 RF trees agree on the sub-family assignment. Note that this does not necessarily mean that the sequence has been correctly classified (according to its database label); in fact, if the assignment was always to same wrong sub-family (in the sense that differs from the database label) the consistency would still be 100%.

**Fig. 3:**
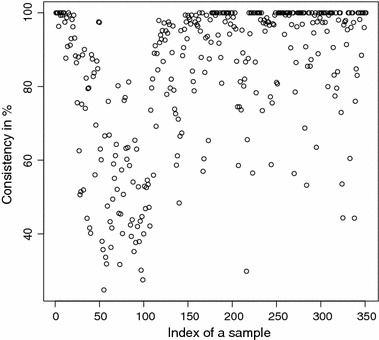
mG consistency values per sequence (AAC). Individual consistency values for mG sequences described by the selected subset of *n*-grams using AAC. The horizontal axis only describes the position of the sequence in the GPCRdb extracted dataset

**Fig. 4:**
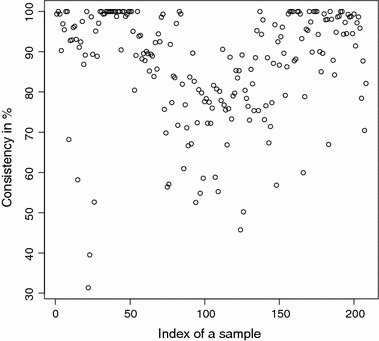
GB consistency values per sequence (AAC). Individual consistency values for GB sequences described by the selected subset of *n*-grams using AAC. The horizontal axis only describes the position of the sequence in the GPCRdb extracted dataset

**Fig. 5:**
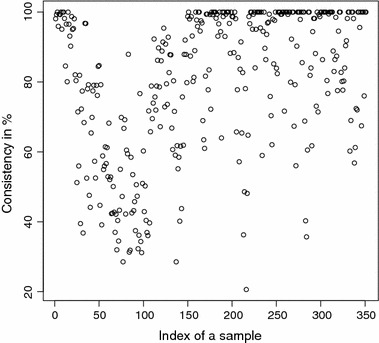
mG consistency values per sequence (SEZ). Individual consistency values for mG sequences described by the Sezerman transformation. The horizontal axis only describes the position of the sequence in the GPCRdb extracted dataset

**Fig. 6:**
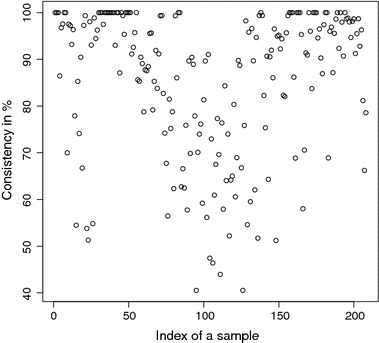
GB consistency values per sequence (SEZ). Individual consistency values for GB sequences described by the Sezerman transformation. The horizontal axis only describes the position of the sequence in the GPCRdb extracted dataset

**Fig. 7:**
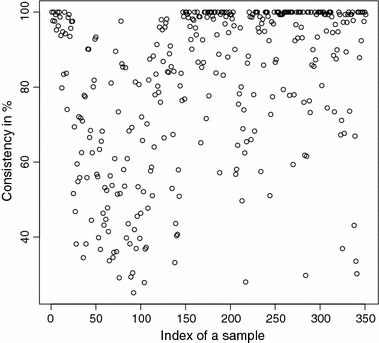
mG consistency values per sequence (DAV). Individual consistency values for mG sequences described by the Davies transformation. The horizontal axis only describes the position of the sequence in the GPCRdb extracted dataset

**Fig. 8:**
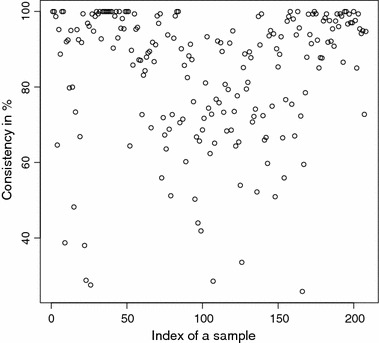
GB consistency values per sequence (DAV). Individual consistency values for GB sequences described by the Davies transformation. The horizontal axis only describes the position of the sequence in the GPCRdb extracted dataset

**Fig. 9:**
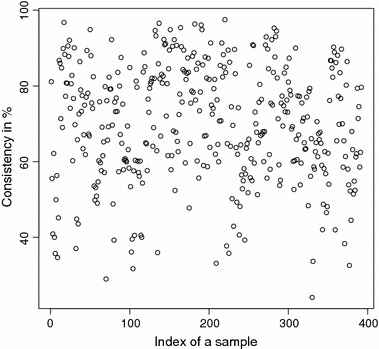
Ph consistency values per sequence (AAC). Individual consistency values for Ph sequences described by the selected subset of *n*-grams using AAC. The horizontal axis only describes the position of the sequence in the GPCRdb extracted dataset

**Fig. 10:**
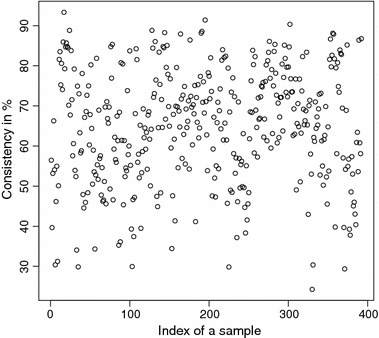
Ph consistency values per sequence (SEZ). Individual consistency values for Ph sequences described by the Sezerman transformation. The horizontal axis only describes the position of the sequence in the GPCRdb extracted dataset

**Fig. 11:**
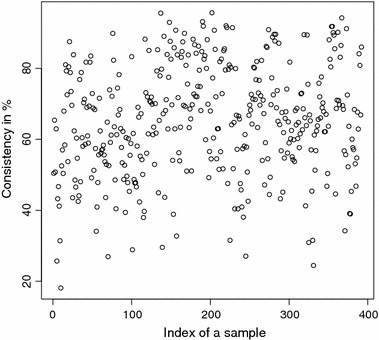
Ph consistency values per sequence (DAV). Individual consistency values for Ph sequences described by the Davies transformation. The horizontal axis only describes the position of the sequence in the GPCRdb extracted dataset

The detailed consistencies for all mG and GB sequences for the subset generated using AAC are, in turn, displayed in Figs. 3 and 4; for the subset using the Sezerman transformation in Figs. 5 and 6; and for the subset using the Davies transformation in Figs. 7 and 8. As an illustration of a sub-family with poor discrimination behavior, similar figures are shown for Ph (see, in turn, Figs. 9, 10, 11). Results for the rest of sub-families can be found in Additional file 1.

From the point of view of database quality assessment and control, we are most interested in those Class C sequences that are not just very frequently misclassified from the point of view of their true (according to the database) class, but also very consistently misclassified to a given *wrong* sub-family by the majority of trees in the RF.

Shortlists of this type of sequences and values of the consistencies of their misclassification were obtained for all Class C sub-families. The criterion for inclusion is that the consistency for the true class (according to database label) is lower than 1/7, meaning that the consistency for some other class must be higher than 1/7. More restrictive criteria might be applied should the expert decide to restrict the data quality control procedure.

Again, we first illustrate this selection using mG, GB and Ph for each of the three transformations. Results can be found in Tables 8, 9 and 10. It would also be important to discount the possible impact of the type of data transformation on the misclassification consistency behavior. For that, we show in Tables 11 and 12, in turn, the sequences shortlisted in at least two out of three transformations and in all three transformations. Similar results for the rest of sub-families can be found in Additional file 2.

**Table 8:** General shortlist of mG (top list), GB (middle list) and Ph (bottom list) sequences from the subset generated using AAC that were consistently misclassified to a different specific sub-family

Name	*mG*						
	*mG*	CS	GB	VN	Ph	Od	Ta
a8dz71_danre	0.045	0.035	0.000	0.205	*0.370*	0.330	0.015
a8dz72_danre	0.028	0.045	0.006	0.198	*0.435*	0.209	0.079
q5i5d4_9tele	0.055	0.011	0.022	0.088	0.203	*0.599*	0.022
q5i5c3_9tele	0.032	0.005	0.000	0.074	0.159	*0.725*	0.005
XP_002735016	0.026	0.000	*0.958*	0.000	0.016	0.000	0.000

Name	*GB*						
	mG	CS	*GB*	VN	Ph	Od	Ta
XP_002738008	*0.784*	0.011	0.086	0.011	0.086	0	0.022

Name	*Ph*						
	mG	CS	GB	VN	*Ph*	Od	Ta
a7sdg9_nemve	*0.836*	0.011	0.063	0.005	0.058	0.011	0.016
b3s157_triad	*0.670*	0.017	0.067	0.095	0.095	0.034	0.022
b3s609_triad	*0.455*	0.037	0.175	0.048	0.101	0.016	0.169
XP_002731604	*0.519*	0.021	0.270	0.026	0.111	0.016	0.037
XP_002732067	*0.613*	0.058	0.145	0.029	0.116	0.023	0.017
XP_001521044	*0.401*	0.006	0.271	0.051	0.141	0.023	0.107
q9pwe1_ictpu	*0.825*	0.005	0.057	0.010	0.103	0.000	0.000
b0uyj3_danre	*0.877*	0.000	0.005	0.000	0.118	0.000	0.000
XP_001075542	0.000	0.006	0.000	0.161	0.122	*0.706*	0.006
XP_001521075	*0.549*	0.006	0.429	0.000	0.017	0.000	0.000

Values in italics are the highest for the specified sequence

**Table 9:** General shortlist of mG (top list), GB (middle list) and Ph (bottom list) sequences from the subset generated using Sezerman transformation that were consistently misclassified to a different specific sub-family

Name	*mG*						
	*mG*	CS	GB	VN	Ph	Od	Ta
a8dz71_danre	0.016	0.011	0.000	0.216	*0.465*	0.270	0.022
a8dz72_danre	0.103	0.034	0.011	0.069	*0.497*	0.194	0.091
q5i5d4_9tele	0.034	0.028	0.000	0.101	0.235	*0.598*	0.006
q5i5c3_9tele	0.033	0.022	0.011	0.116	0.254	*0.547*	0.017
XP_002163014	0.119	0.017	0.028	*0.435*	0.294	0.079	0.028

Name	*GB*						
	mG	CS	*GB*	VN	Ph	Od	Ta
b3rj55_triad	*0.466*	0.052	0.110	0.084	0.194	0.010	0.084
XP_002738008	*0.574*	0.024	0.136	0.077	0.118	0.012	0.059

Name	*Ph*						
	mG	CS	GB	VN	*Ph*	Od	Ta
a7sdg9_nemve	*0.615*	0.046	0.126	0.057	0.126	0.006	0.023
a7s0d2_nemve	*0.591*	0.069	0.103	0.039	0.128	0.034	0.034
b3s157_triad	*0.706*	0.011	0.068	0.056	0.079	0.000	0.079
q4spr3_tetng	*0.280*	0.065	*0.480*	0.010	0.120	0.005	0.040
NP_001093020	0.022	0.005	0.102	*0.699*	0.140	0.027	0.005
b0uyj3_danre	*0.870*	0.005	0.041	0.010	0.073	0.000	0.000
XP_001075542	0.030	0.015	0.005	0.197	0.099	*0.611*	0.044
XP_001521075	*0.430*	0.006	*0.436*	0.017	0.087	0.000	0.023

Values in italics are the highest for the specified sequence

**Table 10:** General shortlist of mG (top list), GB (middle list) and Ph (bottom list) sequences from the subset generated using Davies transformation that were consistently misclassified to a different specific sub-family

Name	*mG*						
	*mG*	CS	GB	VN	Ph	Od	Ta
a8dz71_danre	0.022	0.043	0.016	*0.239*	*0.342*	*0.299*	0.038
a8dz72_danre	0.006	0.017	0.000	0.115	0.293	*0.557*	0.011
q5i5c3_9tele	0.124	0.006	0.006	0.102	0.085	*0.667*	0.011
a7s0d3_nemve	0.108	0.072	0.006	0.174	*0.419*	0.186	0.036
b3s5y8_triad	0.059	0.000	*0.909*	0.016	0.005	0.000	0.011
XP_002187232	0.117	0.000	*0.766*	0.006	0.070	0.006	0.035

Name	*GB*						
	mG	CS	*GB*	VN	Ph	Od	Ta
a7s6r9_nemve	*0.683*	0.032	0.063	0.069	0.111	0.005	0.037
b3rj55_triad	*0.559*	0.028	0.107	0.085	0.153	0.006	0.062
XP_002738008	*0.560*	0.010	0.015	0.210	0.120	0.055	0.030
a8q0q5_bruma	*0.566*	0.032	0.111	0.127	0.116	0.011	0.037

Name	*Ph*						
	mG	CS	GB	VN	*Ph*	Od	Ta
a7sdg9_nemve	*0.564*	0.069	0.112	0.106	0.117	0.005	0.027
a7s1x6_nemve	*0.697*	0.022	0.059	0.059	0.119	0.005	0.038
a7s0d2_nemve	*0.556*	0.012	0.175	0.058	0.135	0.053	0.012
b3s157_triad	*0.674*	0.032	0.128	0.021	0.080	0.000	0.064
XP_002940870	*0.269*	0.183	0.059	*0.274*	0.118	0.016	0.081
XP_002941708	*0.589*	0.044	0.039	0.100	0.139	0.000	0.089
NP_001093018	0.000	0.000	0.000	*0.906*	0.094	0.000	0.000
NP_001093016	0.010	0.000	0.000	*0.907*	0.074	0.005	0.005
NP_001093017	0.006	0.039	0.011	*0.782*	0.134	0.006	0.022
XP_002936172	0.000	0.000	0.000	*0.895*	0.074	0.032	0.000
XP_684341	*0.517*	0.095	0.060	0.109	0.090	0.015	0.114
q9pwe1_ictpu	*0.923*	0.000	0.000	0.012	0.053	0.000	0.012
XP_001075542	0.027	0.000	0.000	0.212	0.125	*0.625*	0.011
XP_001521075	*0.305*	0.021	*0.574*	0.005	0.068	0.016	0.011

Values in italics are the highest for the specified sequence

**Table 11:** Refined shortlist of mG (top list), GB (middle list) and Ph (bottom list) sequences from the subset generated using the three transformations that were consistently misclassified to a different specific sub-family in *two out of the three* transformations

Name	*mG*						
	*mG*	CS	GB	VN	Ph	Od	Ta
q5i5d4_9tele	0.055	0.011	0.022	0.088	0.203	*0.599*	0.022

Name	*GB*						
	mG	CS	*GB*	VN	Ph	Od	Ta
b3rj55_triad	*0.559*	0.028	0.107	0.085	0.153	0.006	0.062

Name	*Ph*						
	mG	CS	GB	VN	*Ph*	Od	Ta
b3s157_triad	*0.670*	0.017	0.067	0.095	0.095	0.034	0.022
q9pwe1_ictpu	*0.825*	0.005	0.057	0.010	0.103	0.000	0.000
b0uyj3_danre	*0.877*	0.000	0.005	0.000	0.118	0.000	0.000

Values in italics are the highest for the specified sequence

**Table 12:** Refined shortlist of mG (top list), GB (middle list) and Ph (bottom list) sequences from the subset generated using the three transformations that were consistently misclassified to a different specific sub-family in *all three* transformations

Name	*mG*						
	*mG*	CS	GB	VN	Ph	Od	Ta
a8dz71_danre	0.045	0.035	0.000	0.205	*0.370*	0.330	0.015
a8dz72_danre	0.028	0.045	0.006	0.198	*0.435*	0.209	0.079
q5i5c3_9tele	0.032	0.005	0.000	0.074	0.159	*0.725*	0.005

Name	*GB*						
	mG	CS	*GB*	VN	Ph	Od	Ta
XP_002738008	*0.784*	0.011	0.086	0.011	0.086	0	0.022

Name	*Ph*						
	mG	CS	GB	VN	*Ph*	Od	Ta
a7sdg9_nemve	*0.836*	0.011	0.063	0.005	0.058	0.011	0.016
b3s157_triad	*0.670*	0.017	0.067	0.095	0.095	0.034	0.022
XP_001075542	0.000	0.006	0.000	0.161	0.122	*0.706*	0.006
XP_001521075	*0.549*	0.006	0.429	0.000	0.017	0.000	0.000

Values in italics are the highest for the specified sequence

### Discussion

As stated in the introduction, the target of the study is not the assessment of the overall classification accuracy that could be obtained for the different sub-families and for each type of data transformation. Nevertheless, such global accuracy was calculated for the three data transformations and reported in the previous section. Accuracies are fairly similar and all in the area of 90%, a result that is consistent with those reported in previous studies [26]. These results suggest that no transformation provides a clear advantage over the others in terms of overall Class C sub-family discrimination.

The confusion matrices in Tables 3, 4 and 5 yield several clear and more specific messages. First, the RF model is shown to have adequate overall discrimination capabilities for all data transformations. This classification, though, is not homogeneous across sub-families; mG, CS and GB are very well discriminated in all data transformations, while Ta is extremely well-discriminated in all data transformations and slightly less so when using Davies’. On the opposite extreme, Od is very poorly classified in all cases. Despite similarities for all data transformations, the selection of *n*-grams using AAC shows some advantage over the more parsimonious Sezerman and Davies transformations.

Inspecting these matrices in more detail, some other interesting patterns emerge: some sub-families do not show clear “preference” in their misclassification, namely mG, CS, GB and Ta; whereas VN, Ph and Od seem to mostly restrict misclassifications to happen between them. VN is mostly misclassified as Ph, while Ph is mostly misclassified as VN (and to a lesser extent to mG); in turn, Od is mostly misclassified as Ph and, to a lesser extent, as VN. Overall, the Ph sub-family seems to take a central role in this misclassification pattern, overlapping the other two sub-families.

The latter results are corroborated by the per-sub-family consistency means reported in Table 6 (and by the means corresponding to the rest of sub-families, not reported here), which show a sizeable overlapping between the Ph and VN sub-families.

Yet again, even if the overall results for each of the sub-families are relevant on their own right, the detailed consistency values per sequence for these sub-families are the key objective of the current study. Corroborating the overall findings, a sizeable proportion of the consistencies of the individual sequences belonging to mG have either 100% or near 100% consistency values, as seen in Figs. 3, 5 and 7. Nonetheless, the consistencies of many sequences with indices roughly between 50 and 150 fall sharply to values under 50% for all transformations. Note that the horizontal axis in these figures only reflects the position of the specific receptor sequence in the GPCRdb extracted data set and, therefore, these results could perhaps indicate the existence of a differentiated sub-group within this sub-family that would require specific attention. A first inspection of GPCRdb identifiers reveals that only a few of the sequences in positions between 50 and 150 are clearly tagged in the database as belonging to a given mG subtype and they are mostly from subtype 3. Many of them are actually uncharacterized. A similar but more attenuated pattern can be seen in Figs. 4, 6 and 8 for the GB sub-family, which also has two known sub-types (GABA$_{B1}$ and GABA$_{B2}$).

Most of the individual consistencies of the sequences for the rest of sub-families (see Additional file 1) show a more homogeneous distribution, mostly with values between 60 and 90%. Sequences of very low consistencies seem to be quite evenly distributed.

Turning now to the final objective of this paper, which is the creation of shortlists of Class C GPCR sequences that are very consistently misclassified, the results reported in Tables 8, 9 and 10 turn out to be quite revealing. First, and unsurprisingly, a poorly discriminated sub-family such as Ph has many more consistent misclassifications than easier to discriminate sub-families such as mG and GB. These results are corroborated by those of the rest of sub-families, compiled in Additional file 2, where, as extreme opposite cases, many Od sequences are shortlisted, in stark contrast to the almost complete absence of Ta sequences. Also, and interestingly, most of the GB and Ph consistently misclassified sequences were assigned to the mG sub-family regardless of data transformation, whereas the shortlisted mG have been unevenly assigned to GB, VN, Ph and Od. A hypothesis to explain this behavior is that, due to the heterogeneity of the mG sub-family, known to include up to 8 different subtypes, some of these subtypes could be relatively close to GB, VN, Ph, or Od in the space spanned by their sequence transformations. For the rest of sub-families, it is also interesting to discover that most of the consistently misclassified Od sequences are assigned to Ph, corroborating the previous results that indicate the strong overlapping between both families.

For the analysis of the influence of data tranformations on the consistency of misclassifications, the results reported in Tables 11 and 12 for mG, GB and Ph are again revealing. Five sequences were consistently misclassified using two out of the three transformations and as much as another eight appeared in all three shortlists. These are sizeable numbers that reveal that, although some of the misclassifications could be data transformation-related, most are transformation-independent. Focusing only on Table 12, we see that it includes three mG sequences, one GB and four Ph.

The same results for the rest of sub-families, found in Additional file 2, indicate that the consistency of misclassification is most transformation-dependent for the most overlapping sub-families (Od and VN). Ten sequences of those sub-families were included in the refined shortlist of all transformations: two CS, two VN and six Od.

Although the investigation of each of these individual sequences is beyond the scope of this paper, some of them will be discussed here. Sequences *a8dz71_danre* and *a8dz72_danre*, reported in the refined list of Table 12, were, uncharacterized proteins derived from an Ensembl automatic analysis pipeline according to the UniProt database; they have since been deleted from the database. Ensembl characterized them as Class C olfactory receptors and note that our RF model predicted them to belong to the Ph sub-family. Also according to UniProt [27], *q5i5c3_9tele*, in the refined list of Table 12, is the unreviewed putative pheromone receptor CPpr14 (note that the RF assigns it consistently to Od and to Ph as a second choice).

Sequence *q8c0m6_mouse*, a CS in the refined shortlist in Additional file 2, is also an *unreviewed* sequence that should be considered only as preliminary data, according to the UniProt database [28]. Also in Additional file 2, *XP_002740613* and *XP_002940324*, which are, in turn, a CS predicted to be a GB and an Od predicted to be a VN, would require further investigation as their sub-family assignment was automatically derived by computational analysis from an annotated genomic sequence by means of a gene prediction mode from the RefSeq databank [29].

Finally, let us insist again on the fact that the criterion used to create these shortlists is flexible and that less or more restrictive criteria could have been defined. Ultimately, the strictness of this criterion should be set by the database curator. In any case, the characteristics of these individual sequences would be the matter of further investigation, even beyond GPCRdb, in the main international protein databases.

## Conclusion

Class C of GPCRs have established themselves as relevant targets in pharmacology for their role as drug targets, especially in pathologies of the central nervous system. This protein family has a heterogeneous sub-family structure, whose investigation has to be carried out from the primary structure of its members in the almost complete absence of information concerning their complete tertiary and quaternary structures.

The automatic discrimination of these sub-families is a challenging task and it has previously been shown to have clear limits. This study has investigated in some detail the hypothesis that such limits could at least be partially caused by sequence mislabeling, a problem that would fall within the remit of database curation. Mislabeling could be revealed by investigating the consistency of misclassification using ensemble techniques. In this study, we have reported experiments carried out with the RF method, which is naturally suited to the the task of measuring classification consistency with little computational effort.

Different sub-families have been shown to display very different discrimination consistency behaviors. Specific attention has been paid to the individual identification of Class C GPCR sequences that were consistently assigned by the RF base classifiers to sub-families other than their *true* one (i.e., misclassified). Sequences consistently misclassified across data transformations have been singled out in refined shortlists as candidates for further labeling investigation. This type of analysis is meant to provide a data engineering quality control tool that is useful not only for GPCR database curators in particular, but for curators of protein databases in general.

## Additional files

**Additional file 1.** Additional figures for Class C GPCR sub-families CS, VN, Od and Ta. They provide the same information concerningsequence-specific consistencies as Figs. 2, 3, 4, 5, 6, 7, 8, 9 and 10 for the remaining Class C GPCR sub-families CS, VN, Od and Ta.

**Additional file 2.** Additional tables for Class C GPCR sub-families CS, VN, Od and Ta. They provide the same detailed information asTables 7, 8, 9, 10 and 11 for the remaining Class C GPCR sub-families CS, VN, Od and Ta.
