# Supplementary material for: Using random forests for assistance in the curation of G-protein coupled receptor databases
Source: Biomed Eng Online. 2017 Aug 18;16(Suppl 1):75. doi: 10.1186/s12938-017-0357-4 (PMC5568607; doi:10.1186/s12938-017-0357-4)
Supplement: Supplementary file 1 — Additional file 1. Additional figures for Class C GPCR sub-families CS, VN, Od and Ta. They provide the same information concerningsequence-specific consistencies as Figs. 2, 3, 4, 5, 6, 7, 8, 9 and 10 for the remaining Class C GPCR sub-families CS, VN, Od and Ta. [file 12938_2017_357_MOESM1_ESM.pdf]

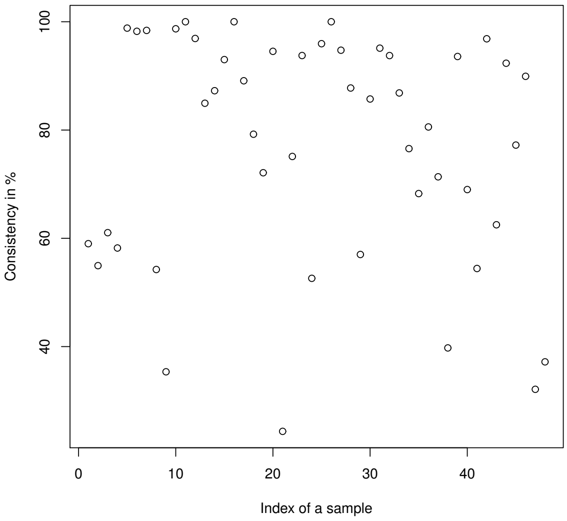

Fig. 1. CS consistency values per sequence (AAC). Individual consistency values for mG sequences described by the selected subset of n-grams using all amino acids. The horizontal axis only describes the position of the sequence in the GPCRdb extracted dataset.

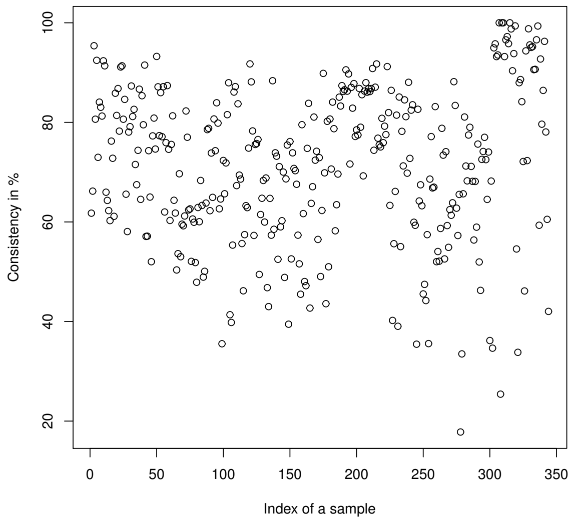

Fig. 2. VN consistency values per sequence (AAC). Individual consistency values for mG sequences described by the selected subset of n-grams using all amino acids. The horizontal axis only describes the position of the sequence in the GPCRdb extracted dataset.

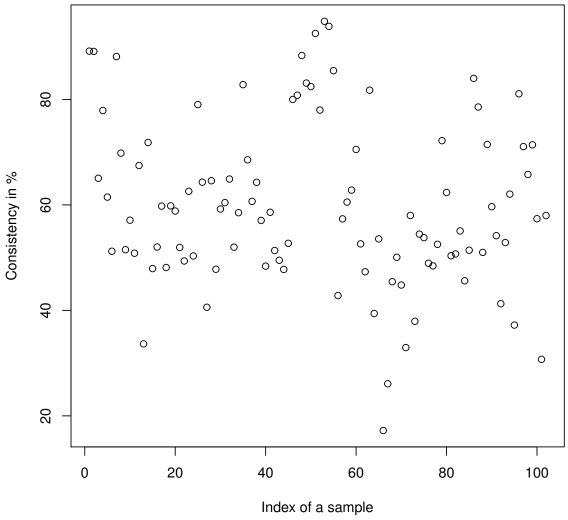

Fig. 3. Od consistency values per sequence (AAC). Individual consistency values for mG sequences described by the selected subset of n-grams using all amino acids. The horizontal axis only describes the position of the sequence in the GPCRdb extracted dataset.

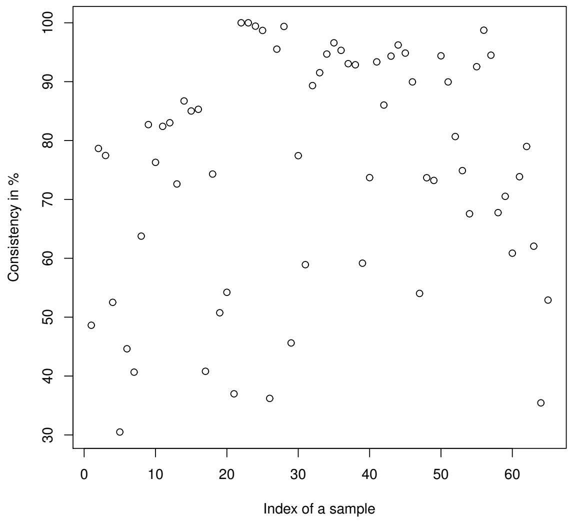

Fig. 4. Ta consistency values per sequence (AAC). Individual consistency values for mG sequences described by the selected subset of n-grams using all amino acids. The horizontal axis only describes the position of the sequence in the GPCRdb extracted dataset.

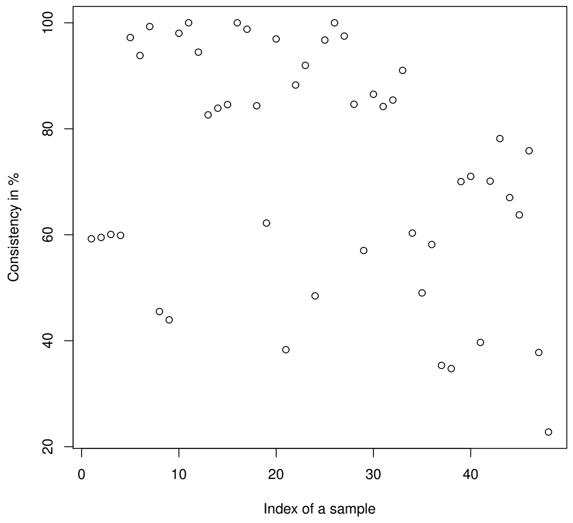

Fig. 5. CS consistency values per sequence (SEZ). Individual consistency values for mG sequences described by the selected subset of n-grams using all amino acids. The horizontal axis only describes the position of the sequence in the GPCRdb extracted dataset.

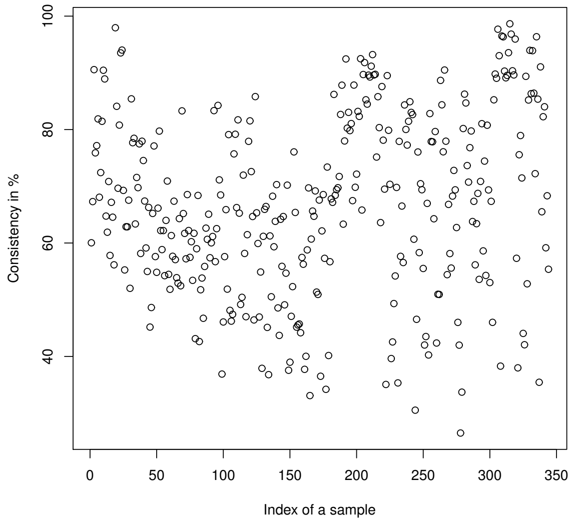

Fig. 6. VN consistency values per sequence (SEZ). Individual consistency values for mG sequences described by the selected subset of n-grams using all amino acids. The horizontal axis only describes the position of the sequence in the GPCRdb extracted dataset.

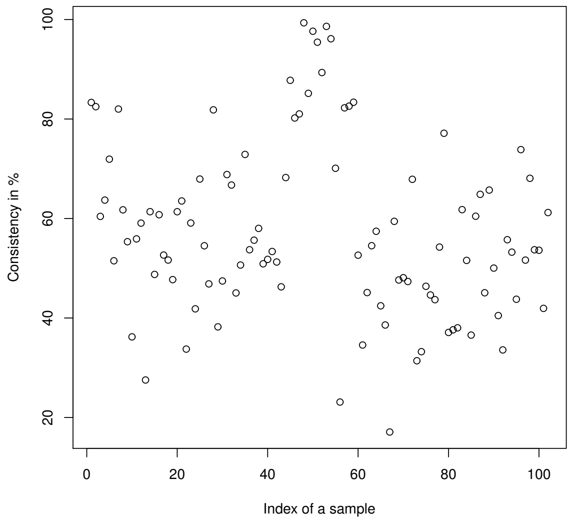

Fig. 7. Od consistency values per sequence (SEZ). Individual consistency values for mG sequences described by the selected subset of n-grams using all amino acids. The horizontal axis only describes the position of the sequence in the GPCRdb extracted dataset.

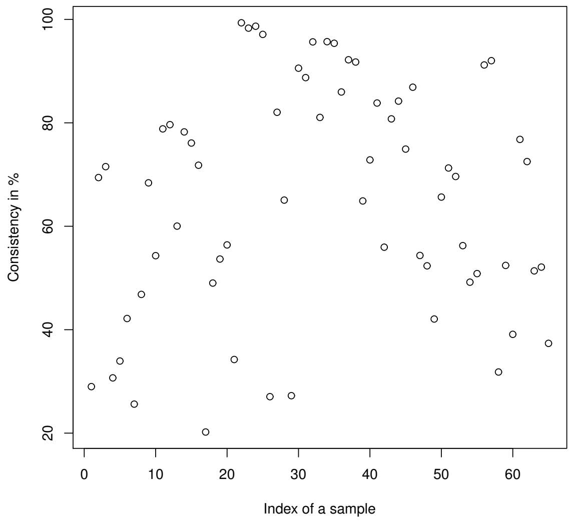

Fig. 8. Ta consistency values per sequence (SEZ). Individual consistency values for mG sequences described by the selected subset of n-grams using all amino acids. The horizontal axis only describes the position of the sequence in the GPCRdb extracted dataset.

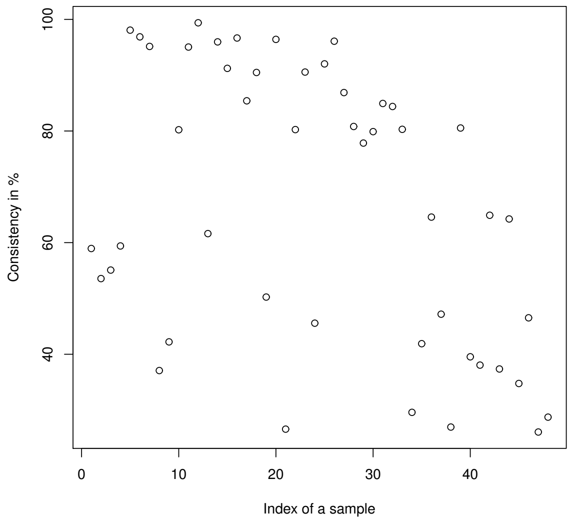

Fig. 9. CS consistency values per sequence (DAV). Individual consistency values for mG sequences described by the selected subset of n-grams using all amino acids. The horizontal axis only describes the position of the sequence in the GPCRdb extracted dataset.

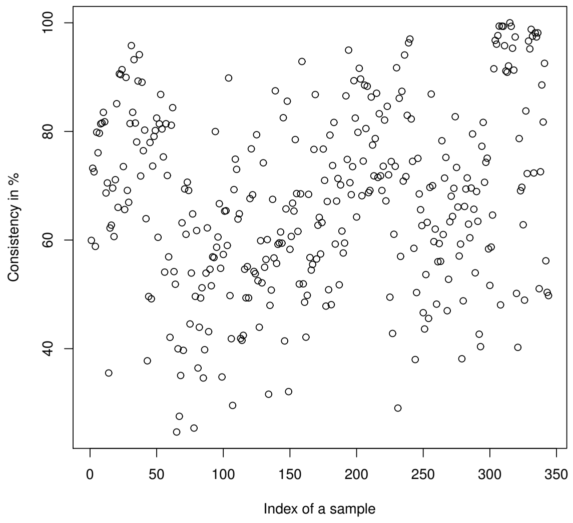

Fig. 10. VN consistency values per sequence (DAV). Individual consistency values for mG sequences described by the selected subset of n-grams using all amino acids. The horizontal axis only describes the position of the sequence in the GPCRdb extracted dataset.

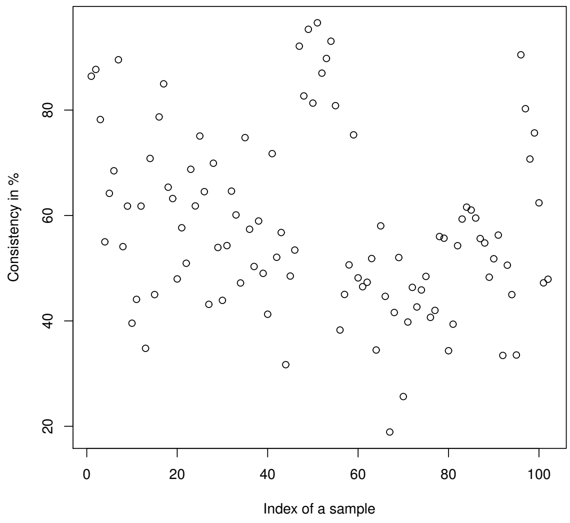

Fig. 11. Od consistency values per sequence (DAV). Individual consistency values for mG sequences described by the selected subset of n-grams using all amino acids. The horizontal axis only describes the position of the sequence in the GPCRdb extracted dataset.

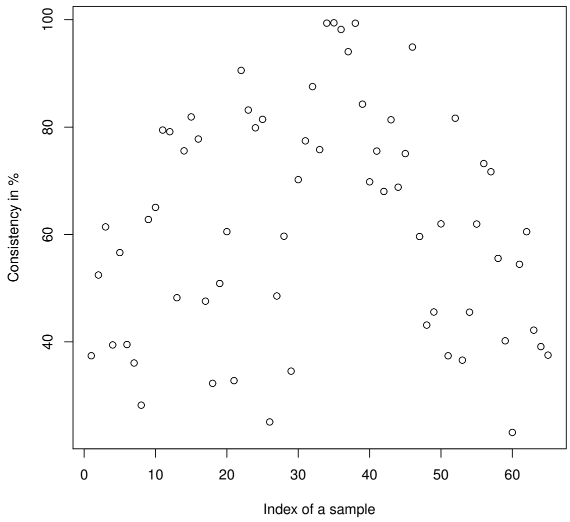

Fig. 12. Ta consistency values per sequence (DAV). Individual consistency values for mG sequences described by the selected subset of n-grams using all amino acids. The horizontal axis only describes the position of the sequence in the GPCRdb extracted dataset.
