# Supplementary material for: Using random forests for assistance in the curation of G-protein coupled receptor databases
Source: Biomed Eng Online. 2017 Aug 18;16(Suppl 1):75. doi: 10.1186/s12938-017-0357-4 (PMC5568607; doi:10.1186/s12938-017-0357-4)
Supplement: Supplementary file 2 — Additional file 2. Additional tables for Class C GPCR sub-families CS, VN, Od and Ta. They provide the same detailed information asTables 7, 8, 9, 10 and 11 for the remaining Class C GPCR sub-families CS, VN, Od and Ta. [file 12938_2017_357_MOESM2_ESM.pdf]

Table 1: General shortlist of sequences of the remaining sub-families not covered in the main text of the paper (CS, VN, Od and Ta), from the subset generated using all amino acids, that were consistently misclassified to a different specific sub-family.

**CS**							
Name	mG	**CS**	GB	VN	Ph	Od	Ta
q8c0m6_mouse	0.265	0.097	**0.584**	0.005	0.027	0.016	0.005
XP_002740613	**0.401**	0.015	0.046	0.086	0.259	0.147	0.046
**VN**							
Name	mG	CS	GB	**VN**	Ph	Od	Ta
q8bid7_mouse	0.032	0.005	0.011	0.085	0.176	**0.660**	0.032
NP_001093066	0.090	0.032	0.042	0.042	0.148	0.005	**0.640**
o70411_rat	0.006	0.012	0.000	0.139	0.162	**0.682**	0.000
o35200_mouse	**0.403**	0.043	0.183	0.048	0.156	0.048	0.118
**Od**							
Name	mG	CS	GB	VN	Ph	**Od**	Ta
o93553_carau	0.006	0.006	0.000	0.281	**0.590**	0.112	0.006
q6unx3_ictpu	0.067	0.017	0.000	0.235	**0.525**	0.128	0.028
gpc6a_human	**0.500**	0.061	0.056	0.094	0.161	0.056	0.072
d1lwx7_sacko	**0.308**	0.151	0.081	0.122	**0.238**	0.058	0.041
XP_002936177	0.059	0.010	0.010	**0.348**	**0.412**	0.142	0.020
XP_002936183	**0.260**	0.045	0.079	**0.215**	**0.333**	0.051	0.017
XP_002940477	0.017	0.000	0.006	0.282	**0.541**	0.133	0.022
XP_002940566	0.027	0.000	0.133	**0.330**	**0.468**	0.043	0.000
XP_002940324	0.079	0.040	0.000	**0.520**	**0.316**	0.034	0.011
gpc6a_danre	**0.244**	0.034	0.040	**0.341**	**0.284**	0.045	0.011
XP_001333050	0.144	0.101	0.034	0.221	0.351	0.139	0.010
**Ta**							
Name	mG	CS	GB	VN	Ph	Od	**Ta**
None							

Table 2: General shortlist of sequences of the remaining sub-families not covered in the main text of the paper (CS, VN, Od and Ta), from the subset generated using the Sezerman transformation, that were consistently misclassified to a different specific sub-family.

			**CS**				
Name	mG	**CS**	GB	VN	Ph	Od	Ta
XP_002123664	**0.370**	0.109	0.024	0.142	**0.313**	0.038	0.005
q8c0m6_mouse	0.194	0.039	**0.611**	0.028	0.083	0.017	0.028
XP_002933302	0.146	0.056	0.022	0.236	**0.416**	0.107	0.017
XP_002740613	**0.293**	0.043	0.048	0.176	0.181	0.122	0.138
			**VN**				
Name	mG	CS	GB	**VN**	Ph	Od	Ta
q8bid7_mouse	0.033	0.011	0.000	0.089	0.094	**0.744**	0.028
XP_002936197	0.018	0.000	0.000	0.094	**0.853**	0.029	0.006
XP_002941777	**0.287**	0.042	0.126	0.126	**0.383**	0.030	0.006
NP_001093066	**0.268**	0.045	0.045	0.121	**0.333**	0.051	0.136
q8tdu1_human	**0.355**	0.089	**0.166**	0.053	0.107	0.095	**0.136**
o35200_mouse	**0.440**	0.038	0.065	0.141	0.201	0.065	0.049
			**Od**				
Name	mG	CS	GB	VN	Ph	**Od**	Ta
gpc6a_human	0.150	0.139	0.021	0.203	**0.294**	0.091	0.102
b3rud8_triad	**0.464**	0.045	0.078	0.145	0.128	0.073	0.067
d1lwx7_sacko	**0.251**	0.121	0.045	0.161	0.146	0.101	0.176
XP_002936183	0.049	0.074	0.049	0.245	**0.510**	0.069	0.005
XP_002940477	0.067	0.012	0.018	0.242	**0.497**	0.121	0.042
XP_002940566	0.025	0.000	0.208	0.269	**0.442**	0.046	0.010
XP_002940324	0.029	0.033	0.000	**0.548**	**0.295**	0.076	0.019
XP_002940329	0.005	0.000	0.000	**0.797**	0.110	0.077	0.011
XP_002941572	0.186	0.017	0.062	**0.311**	**0.356**	0.068	0.000
XP_002942058	0.011	0.017	0.000	0.303	**0.612**	0.051	0.006
XP_002941773	0.060	0.012	0.006	0.353	**0.485**	0.084	0.000
gpc6a_danre	**0.401**	0.042	0.000	0.102	**0.359**	0.060	0.036
			**Ta**				
Name	mG	CS	GB	VN	Ph	Od	**Ta**
			None				

Table 3: General shortlist of sequences of the remaining sub-families not covered in the main text of the paper (CS, VN, Od and Ta), from the subset generated using the Davies transformation, that were consistently misclassified to a different specific sub-family.

			**CS**				
Name	mG	**CS**	GB	VN	Ph	Od	Ta
q8c0m6_mouse	0.066	0.022	**0.809**	0.038	0.038	0.011	0.016
XP_002740613	**0.320**	0.017	0.166	0.099	**0.249**	0.088	0.061
			**VN**				
Name	mG	CS	GB	**VN**	Ph	Od	Ta
XP_002937449	**0.663**	0.023	0.091	0.109	0.097	0.011	0.006
XP_002943694	**0.527**	0.011	0.176	0.082	0.143	0.011	0.049
NP_001093066	**0.417**	0.073	0.057	0.036	0.255	0.031	0.130
NP_001093039	0.033	0.050	0.011	0.094	**0.561**	0.150	0.100
a0t300_danre	0.066	0.013	0.007	0.112	**0.408**	**0.382**	0.013
q8tdu1_human	0.047	0.036	0.005	0.109	0.130	**0.641**	0.031
o70413_rat	0.039	0.182	0.011	0.127	0.171	**0.431**	0.039
o35200_mouse	**0.431**	0.006	0.221	0.110	0.182	0.039	0.011
			**Od**				
Name	mG	CS	GB	VN	Ph	**Od**	Ta
a3qjy1_danre	0.022	0.016	0.000	0.038	**0.809**	0.104	0.011
a0t301_danre	0.029	0.017	0.023	0.194	**0.589**	0.126	0.023
a3qjy5_danre	0.005	0.011	0.000	0.107	**0.717**	0.139	0.021
XP_001332817	0.080	0.150	0.015	0.140	**0.465**	0.095	0.055
gpc6a_human	0.148	0.021	0.042	**0.238**	**0.418**	0.111	0.021
b3rud8_triad	**0.487**	0.010	0.194	0.141	0.136	0.010	0.021
d1lwx7_sacko	0.143	**0.235**	0.184	0.102	0.214	0.077	0.046
XP_002936177	0.174	0.140	0.017	0.191	**0.315**	0.073	0.090
XP_002936183	0.113	0.005	0.097	0.290	**0.409**	0.070	0.016
XP_002940477	0.021	0.021	0.000	0.245	**0.511**	0.133	0.069
XP_002940566	0.071	0.033	**0.443**	0.197	0.202	0.055	0.000
XP_002940324	0.044	0.025	0.015	**0.529**	**0.358**	0.025	0.005
XP_002940329	0.011	0.000	0.000	**0.466**	**0.429**	0.090	0.005
XP_002941572	0.071	0.016	0.005	0.191	**0.585**	0.104	0.027
XP_002941773	0.034	0.017	0.017	0.191	**0.652**	0.067	0.022
			**Ta**				
Name	mG	CS	GB	VN	Ph	Od	**Ta**
q4rx46_tetng	**0.620**	0.070	0.048	0.053	0.059	0.011	0.139
a4phq8_danre	0.021	0.042	0.005	0.174	**0.521**	0.168	0.068

Table 4: Refined shortlist of sequences of the remaining sub-families not covered in the main text of the paper (CS, VN, Od and Ta), from the subset generated using the three transformations, that were consistently misclassified to a different specific sub-family in *two out of the three* transformations.

			**CS**				
Name	mG	**CS**	GB	VN	Ph	Od	Ta
			**None**				
			**VN**				
Name	mG	CS	GB	**VN**	Ph	Od	Ta
q8bid7_mouse	0.032	0.005	0.011	0.085	0.176	**0.660**	0.032
q8tdu1_human	0.047	0.036	0.005	0.109	0.130	**0.641**	0.031
			**Od**				
Name	mG	CS	GB	VN	Ph	**Od**	Ta
XP_002936177	0.059	0.010	0.010	**0.348**	**0.412**	0.142	0.020
gpc6a_danre	0.244	0.034	0.040	**0.341**	0.284	0.045	0.011
b3rud8_triad	**0.464**	0.045	0.078	0.145	0.128	0.073	0.067
XP_002940329	0.011	0.000	0.000	0.466	0.429	0.090	0.005
XP_002941572	0.071	0.016	0.005	0.191	**0.585**	0.104	0.027
XP_002941773	0.034	0.017	0.017	0.191	**0.652**	0.067	0.022
			**Ta**				
Name	mG	CS	GB	VN	Ph	Od	**Ta**
			None				

Table 5: Refined shortlist of sequences of the remaining sub-families not covered in the main text of the paper (CS, VN, Od and Ta), from the subset generated using the three transformations, that were consistently misclassified to a different specific sub-family in *all three* transformations.

			**CS**				
Name	mG	**CS**	GB	VN	Ph	Od	Ta
q8c0m6_mouse	0.265	0.097	**0.584**	0.005	0.027	0.016	0.005
XP_002740613	**0.401**	0.015	0.046	0.086	0.259	0.147	0.046
			**VN**				
Name	mG	CS	GB	**VN**	Ph	Od	Ta
NP_001093066	0.090	0.032	0.042	0.042	0.148	0.005	**0.640**
o35200_mouse	**0.403**	0.043	0.183	0.048	0.156	0.048	0.118
			**Od**				
Name	mG	CS	GB	VN	Ph	**Od**	Ta
gpc6a_human	**0.500**	0.061	0.056	0.094	0.161	0.056	0.072
d1lwx7_sacko	**0.308**	0.151	0.081	0.122	**0.238**	0.058	0.041
XP_002936183	**0.260**	0.045	0.079	**0.215**	**0.333**	0.051	0.017
XP_002940477	0.017	0.000	0.006	0.282	**0.541**	0.133	0.022
XP_002940566	0.027	0.000	0.133	**0.330**	**0.468**	0.043	0.000
XP_002940324	0.079	0.040	0.000	**0.520**	**0.316**	0.034	0.011
			**Ta**				
Name	mG	CS	GB	VN	Ph	Od	**Ta**
			None				
